# Supplementary material for: Label-Free Saliva Test for Rapid Detection of Coronavirus Using Nanosensor-Enabled SERS
Source: Bioengineering (Basel). 2023 Mar 22;10(3):391. doi: 10.3390/bioengineering10030391 (PMC10045265; doi:10.3390/bioengineering10030391)
Supplement: Supplementary file 1 [file bioengineering-10-00391-s001.zip › bioengineering-2237745-supplementary.pdf]

Label-Free Saliva Test for Rapid Detection of Coronavirus Using Nanosensor-Enabled SERS

Swarna Ganesh [1,2,3], Ashok Kumar Dhinakaran [1,2,3], Priyatha Premnath [4] ,Krishnan Venkatakrishnan [1,2,3] and Bo Tan [1,2,5,*]

1 Keenan Research Center for Biomedical Science, Unity Health Toronto, Toronto, ON M5B 1W8, Canada
2 Institute for Biomedical Engineering, Science and Technology (I BEST), Partnership between Toronto Metropolitan University and St. Michael's Hospital, Toronto, ON M5B 1W8, Canada
3 Ultrashort Laser Nanomanufacturing Research Facility, Department of Mechanical and Industrial Engineering, Toronto Metropolitan University, 350 Victoria Street, Toronto, ON M5B 2K3, Canada
4 Department of biomedical engineering, College of Engineering and Applied Sciences, University of Wisconsin, Milwaukee, WI 53211, USA
5 Nanocharacterization Laboratory, Department of Aerospace Engineering, Toronto Metropolitan University, 350 Victoria Street, Toronto, ON M5B 2K3, Canada
* Correspondence: tanbo@torontomu.ca

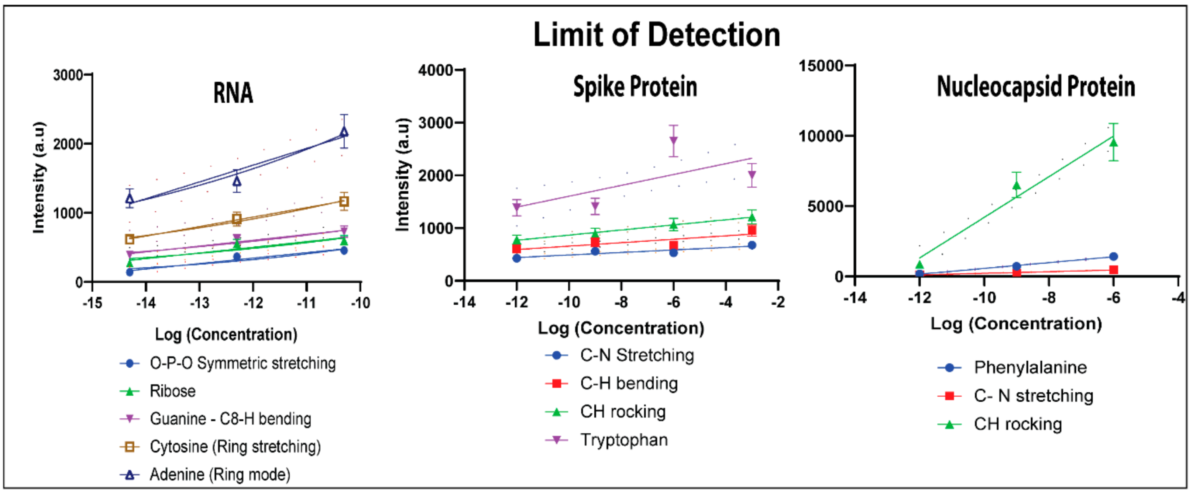

Figure S1. Limit of detection analysis showcasing the linear relationship between the concentration of the viral components and the SERS intensity.

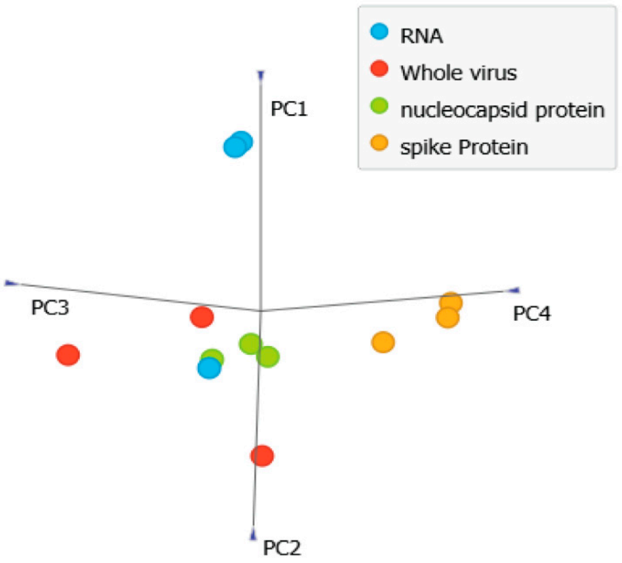

Figure S2. PCA plot showcasing the similarities between individual viral components and the whole virus.

b Fluorescence spectra showcasing the reduction in intensity of characterstic absorption peaks at 547 nm

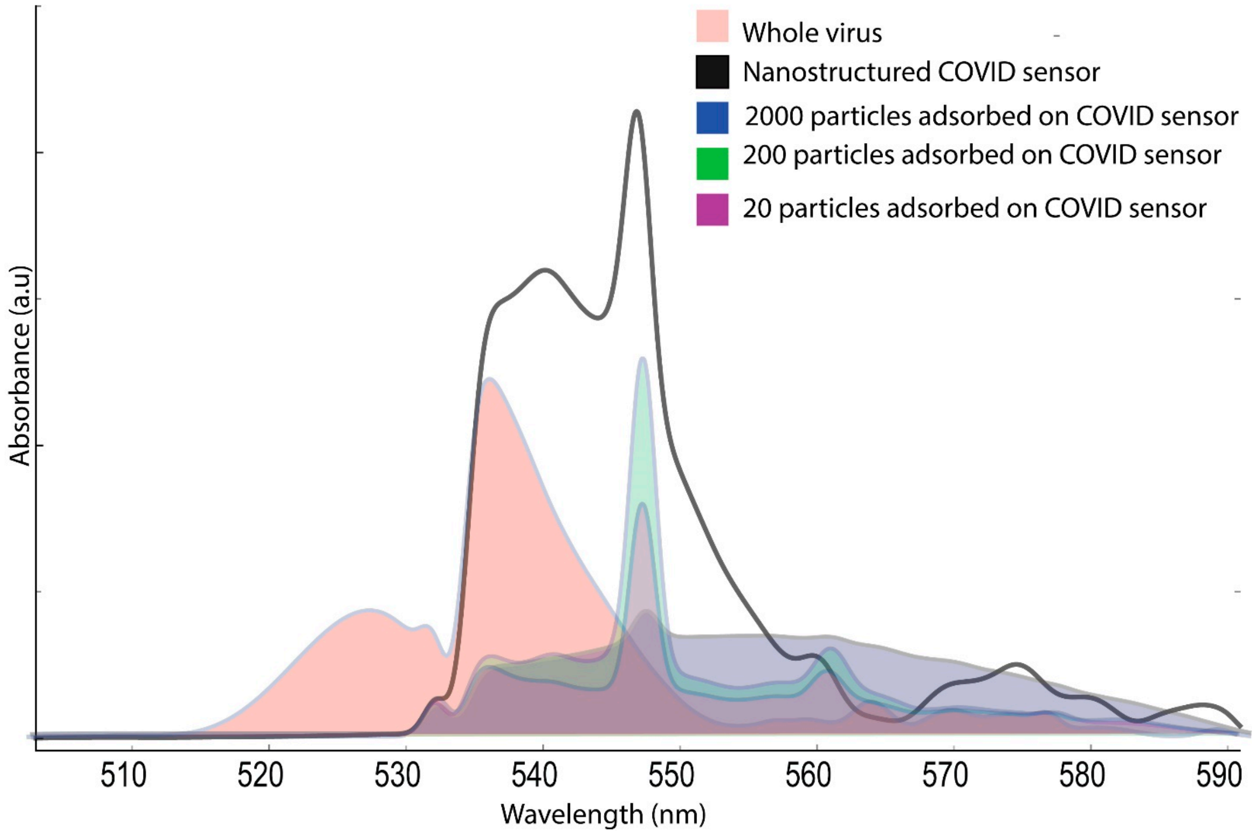

Figure S3. Decrease in the intensity of the characteristic peak of nanostructured COVID sensor at 547 nm on adsorption of whole virus particles indicating efficient charge transfer between nanostructured COVID sensor and the surface of the virus.

Table S1. Table showcasing the solid positive linear correlation between the characteristic peaks of SARS - CoV2 virus components and concentration.

Molecule	Peak Assignments	R^2	*p*-value
RNA	O-P-O Symmetric stretching	0.8814	<0.0001
RNA	Ribose	0.7662	<0.0001
RNA	Guanine - C8-H bending	0.8178	<0.0001
RNA	Cytosine (Ring stretching)	0.8537	<0.0001
RNA	Adenine (Ring mode)	0.7968	<0.0001
Spike Protein	C-N stretching	0.583	<0.0001
Spike Protein	C-H bending	0.5364	0.0002
Spike Protein	CH rocking	0.7214	<0.0001
Spike Protein	tryptophan	0.3901	0.0032
Nucleocapsid Protein	Phenylalanine	0.9622	<0.0001
Nucleocapsid Protein	CH rocking	0.9191	<0.0001
Nucleocapsid Protein	C-N stretching	0.938	<0.0001

Table S2. List of Techniques/ Sensors showing their detection parameters in comparison to our technique.

Technique	Limit of detection	Time	Reference
Multiple antibody assay	1.1×10^5 copies/ml	NA	[47]
RT-PCR	3.8 & 5.2 copies per reaction	NA	[48]
Solsten SARS-CoV-2 Antigen Enzyme Linked ImmunoSorbent Assay Kit	4.1 PFU/mL	NA	[49]
Elecsys SARS-CoV-2 Antigen ElectroChemiLuminescence ImmunoAssay	37 PFU/mL	NA	[49]
Simoa SARS CoV-2 N Protein Advantage Kit	0.15 PFU/mL	NA	[49]
Recombinase polymerase amplification (RPA)	2 copies per sample	50 mins	[50]
Recombinase polymerase amplification (RPA)	2.5 copies/µl input (RNA standard); 1 copy/µl input (pseudovirus)	50 mins	[51]
LAMP (Fluoresence)	30 copies/µL (150 copies)	40 min	[52]
PCR (Lateral flow Assay)	13.5 copies/µL	65 mins	[53]
PER (Fluoresence)	1.3 pM	40 min	[54]
SERS Sensor	10 copies /mL	10 min	This study
